# Neutrophil to lymphocyte ratio (NLR) prognostic effects on heart failure; a systematic review and meta-analysis

**DOI:** 10.1186/s12872-023-03572-6

**Published:** 2023-11-14

**Authors:** Mehrbod Vakhshoori, Sepehr Nemati, Sadeq Sabouhi, Behzad Yavari, Mehrnaz Shakarami, Niloofar Bondariyan, Sayed Ali Emami, Davood Shafie

**Affiliations:** 1 Heart Failure Research Center, Cardiovascular Research Institute, Isfahan University of Medical Sciences, Isfahan, Iran; 2 School of Medicine, Tehran Azad University of Medical Sciences, Tehran, Iran; 3 Student Research Committee, School of Medicine, Isfahan University of Medical Sciences, Isfahan, Iran; 4 Cardiac Rehabilitation Research Center, Cardiovascular Research Institute, Isfahan University of Medical Sciences, Isfahan, Iran; 5 Department of Clinical Pharmacy, School of Pharmacy, Shiraz University of Medical Sciences, Shiraz, Iran

**Keywords:** Neutrophil to lymphocyte ratio, Heart failure, Prognosis, Mortality, Systematic review, Meta-analysis

## Abstract

**Background:**

Neutrophil to lymphocyte ratio (NLR), as a recent inflammatory index, has been reported to be a prognostic tool in different diseases. However, implication of this ratio in heart failure (HF) is less investigated. In this systematic review and meta-analysis, we aimed to assess the potential impact of NLR on HF clinical outcomes.

**Methods:**

Relevant English published records in PubMed, Scopus, Embase, and Web of Science were screened up to July 2023. Articles reporting clinical outcomes (follow-up or in-hospital mortality, readmission, HF prediction, extended hospital stay length, pulmonary vascular resistance, atrial fibrillation, renal disease and functional capacity) in HF sufferers were collected for further analysis with addition of NLR difference stratified by death/survived and HF status.

**Results:**

Thirty-six articles (*n* = 18231) were finally selected which reported NLR in HF sufferers (mean: 4.38, 95% confidence interval (CI): 4.02–4.73). We found 25 articles reported NLR and total mortality (either follow-up death (*N* = 19): 4.52 (95% CI: 4.03–5.01) or in-hospital death (*N* = 10): 5.33 (95% CI: 4.08–6.57)) with mean NLR of 4.74 (95% CI: 4.28–5.20). NLR was higher among deceased patients compared to survived ones (standard mean difference: 0.67 (95% CI: 0.48–0.87), *P* < 0.001)). NLR was found to be related with higher mortality risk (continuous variable: hazard ratio (HR): 1.12, 95% CI: 1.02–1.23, *P* = 0.013), categorical variable: HR: 1.77, 95% CI: 1.27–2.46, *P* = 0.001, T2 vs. T1: HR:1.56, 95%CI: 1.21–2.00, *P* = 0.001, T3 vs. T1: HR:2.49, 95%CI: 1.85–3.35, *P* < 0.001). Other aforementioned variables were not feasible to analyze due to presence of few studies.

**Conclusions:**

NLR is a simple and acceptable prognostic tool for risk stratification and prioritizing high risk patients in clinical settings, especially in resource limited nations.

**Supplementary Information:**

The online version contains supplementary material available at 10.1186/s12872-023-03572-6.

## Introduction

Heart failure (HF) is commonly considered as the end stage of many cardiovascular diseases (CVDs) [1, 2]. This disorder is simply characterized by inability of cardiac tissue pumping the oxygen and blood to meet the metabolic demands of body organs. HF prevalence is still rising rapidly and it has been estimated to increase by 46% in 2030 [3]. Globally, 64.3 million people suffer from this chronic disease leading to a significant economic burden on healthcare system [4]. For instance, approximately $65 billion has been reported as direct HF management cost for each year [5]. Despite substantial improvement in context of implementing new treatment modalities, HF mortality rate is still concerning [6, 7]. Five-year death rate has been indicated to be 42.3% and only 10% of HF sufferers survive after 10 years post HF diagnosis [4, 8]. Therefore, early diagnosis and appropriate delivery of therapeutic interventions are pivotal steps in HF era.

In addition to several previously proved HF risk factors, one of the major pathways in CVDs pathogenesis is related to inflammation and several biomarkers have been introduced in this regard [9–13]. The inflammatory cytokines lead to cardiac cell apoptosis, fibrosis and consequent adverse ventricular remodeling [14]. Neutrophils and lymphocytes are two main arms of inflammation and division of these two blood indices results in introduction of a recent inflammatory index, named neutrophil to lymphocyte ratio (NLR), which has been reported to be a useful prognostic tool in CVDs [15–17]. Due to an imbalance between inflammatory and anti-inflammatory pathways in HF, neutrophil apoptosis decreases leading to heightened absolute counts and increased rate of HF occurrence [18–20]. On the other hand, decompensated state of HF results in lowering lymphocyte counts and lymphocytopenia has been indicated to be an independent mortality predictor in HF [21]. It seems this simple and inexpensive tool might be prognostic in clinical settings. However, reported data are still controversial. For instance, in Delcea et al. and Davran et al.’s studies, findings were in favor of significant NLR association with HF clinical outcomes [22, 23]. On the other hand, Liu et al. and Pourafkari and colleagues suggested this biomarker might not independently predict HF outcomes. Due to these inconsistencies as well as presence of literature gap, a thorough study is required [24, 25].

In this systematic review and meta-analysis, we aimed to assess the potential effect of NLR on different clinical outcomes among patients with HF.

## Materials and methods

### Protocol registration

We registered current systematic review and meta-analysis in International Prospective Register of Systematic Reviews (PROSPERO) with identification number of CRD42022350800. There was not any protocol deviation in current study. This study was also implemented based on Preferred Reporting Items for Systematic Reviews and Meta-Analyses (PRISMA) guideline [26].

### Inclusion and exclusion criteria

We conducted a systematic review of the literature and evaluated all English peer-reviewed studies that reported the impact of the NLR on clinical outcomes in HF patients. We structured our research assessment using the population, exposure, comparator, outcomes, and study designs (PECOS) framework. In this context, our study focused on patients suffering from HF as the defined population. Exposure and comparator elements were not applicable, as our primary objective was to examine the potential impact of NLR across all HF patients. Regarding the outcomes, we considered several factors, including mortality, rehospitalization, HF prediction, extended hospital stay, pulmonary vascular resistance, atrial fibrillation (AF), progression to renal disease, and functional capacity. For study designs, the inclusion criteria were studies with cross-sectional, case–control, cohort, and randomized clinical trial (RCT) designs. For the exclusion criteria, we discarded meeting abstracts, editorials, case report, case series and any studies with incomplete desired outcome as well as studies on animal species.

### Strategy of literature search

Four well-known electronic medical databases including PubMed, Scopus, Embase and Web of Science were screened up to July 2023. In Scopus database, titles, abstracts and keywords were searched. In PubMed, Embase and Web of Science, titles and abstracts were investigated. We used the following search strategy (using medical subject headings (MeSH) and non-MeSH terms) in all aforementioned databases to collect all relevant records: (“neutrophil* to lymphocyte* ratio” OR “neutrophil *-lymphocyte*” OR “neutrophil*-lymphocyte* ratio” OR “neutrophil* to lymphocyte*” OR “neutrophil*-to-lymphocyte* ratio” OR “neutrophil*-to lymphocyte* ratio” OR “neutrophil* to-lymphocyte* ratio” OR “neutrophil */lymphocyte* ratio” OR “neutrophil*/lymphocyte*” OR “nlr”) AND (“heart failure” OR “cardiac failure” OR “heart insufficiency” OR “cardiac insufficiency” OR “congestive heart failure” OR “congestive cardiac failure” OR “decompensated heart failure” OR “decompensated cardiac failure” OR “decompensated heart insufficiency” OR “decompensated cardiac insufficiency” OR “acute decompensated heart failure” OR “acute decompensated cardiac failure” OR “acute decompensated heart insufficiency” OR “acute decompensated cardiac insufficiency” OR “hf”).

### Selection process

**Fig. 1:**
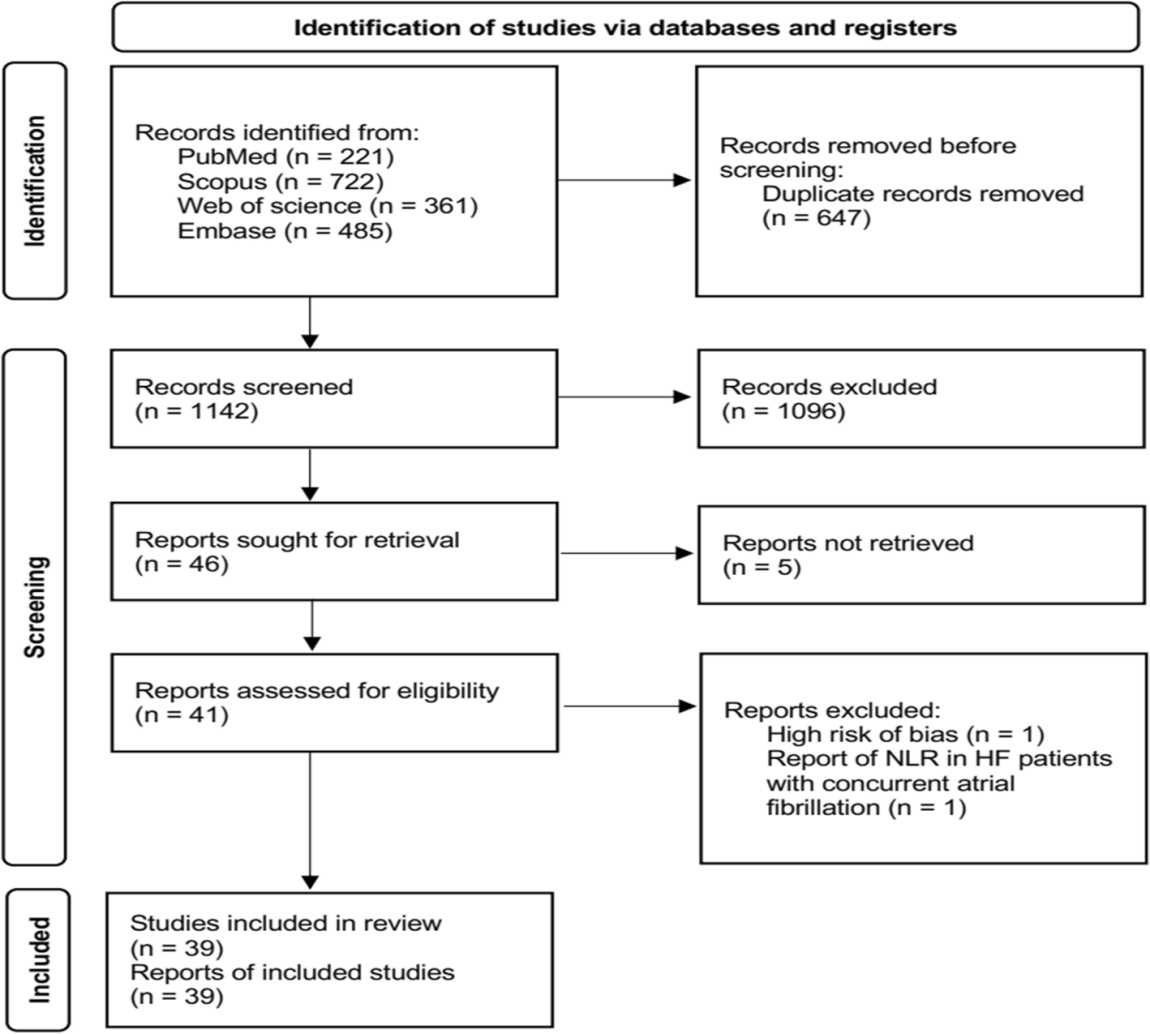
Flow diagram of current study

Three authors (M. V., N. B. and SA. E.) carefully screened titles and abstracts and gathered the full-texts of all relevant articles independently within four aforementioned databases. Only one record was considered in case of duplicated articles. Any disagreement was resolved by consensus. We also provided flow-diagram of current study in Fig. 1.

### Data gathering process

The following items were screened in each recruited record: first author’s name plus publication year, study design, sample size, male frequency, age (mean ± standard deviation (SD) or median (interquartile range (IQR)), as reported), follow-up period (if applicable), NLR (mean ± SD, median (IQR), as reported), NLR tertiles, quartiles and cut-off points (as reported) as well as HF outcomes (mortality (follow-up or in-hospital death), rehospitalization, HF prediction, extended length of hospital stay, pulmonary vascular resistance, AF, progression to renal disease and functional capacity, as reported).

### Risk of bias assessment

In order to evaluate quality and risk of bias in each enrolled article, the following assessment tools were used according to study designs: cross-sectional studies (a critical appraisal tool (AXIS)), case–control studies (national institute of health (NIH) quality assessment tool), cohort studies (Joanna Briggs Institute (JBI) critical appraisal checklist for cohort studies) and RCT (JBI critical appraisal checklists for RCT) [27–30]. We also assessed the certainty of the pre-defined outcomes using Grading of Recommendations Assessment, Development and Evaluation (GRADE) framework.

### Statistical analysis

Pooled effect sizes were provided as mean and hazard ratio (HR) with 95% confidence interval (CI), as appropriate. We used Wan et al.’s and Hozo et al.’s methods to convert median (IQR) and median (range) to mean ± SD for continuous variables, respectively [31, 32]. Cochran’s Q statistic, *I*^*2*^ and tau squared (τ^2^) were used to assess heterogeneity. We used random effects model to implement downstream analyses. Forest plots were depicted to show NLR mean and HR according to studies reported this index in HF subjects. We also provided NLR forest plots according to all-cause mortality and death/survival as well as HF status. In addition to funnel plots, the Egger’s and Begg’s tests as well as Duval and Tweedie’s trim-and-fill method were used to assess heterogeneity and publication bias. Excel datasheet was utilized for data entrance and all analyses were done using comprehensive meta-analysis (CMA) software (version 2.0).

## Results

### Study selection and characteristics

**Fig. 2:**
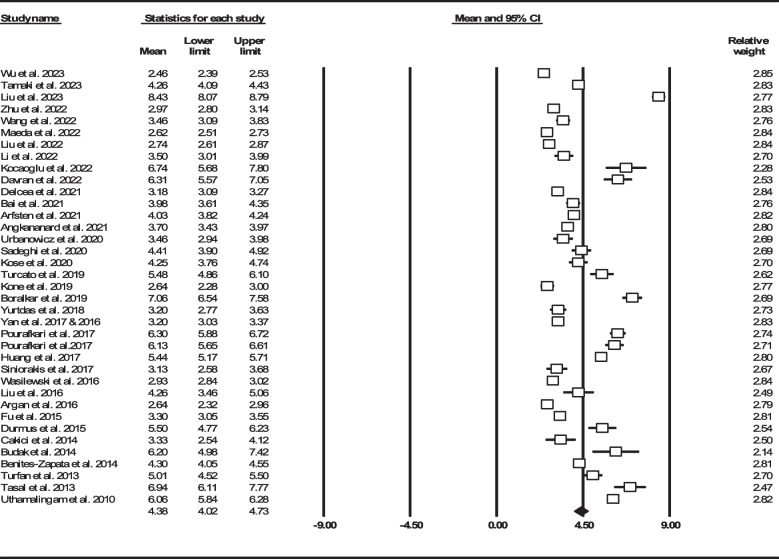
Forest plot for mean NLR based on total study population

After reviewing 1672 articles and elimination of duplicated items as well as other non-relevant articles, we identified 39 articles (*n* = 27256, age: 70.69 ± 13.53 years, 60.41% male) that reported NLR in HF individuals (Fig. 1) [22–25, 33–68]. Two articles on same number of participants with similar outcomes which have been performed by the same authors were considered to be a single record [45, 46]. We provided summary of all recruited studies in Table 1. Three articles were not added to assess mean NLR due to report of this biomarker other than mean ± SD or median (IQR) [34, 40, 68]. Total mean NLR in the remaining 36 studies (*n* = 18231) were found to be 4.38 (95% CI: 4.02–4.73) (Fig. 2).

**Table 1:** Summary of included studies reporting neutrophil to lymphocyte ratio and heart failure clinical outcomes

Authors	Design	Sample size		Male (%)	Age	Follow-up duration	NLR	Outcomes
Wu et al. 2023 [60]	Cross-sectional	Total	1207	689 (57.08)	Mean ± SD: 67.3 ± 12.5	Median (IQR): 66 (35–105.5) months	Mean ± SD: 2.46 ± 1.18 Median (IQR): 2.4(1.7–3.3) NLR quartiles: Q1: < 1.7 Q2: 1.7–2.4 Q3: 2.4–3.3 Q4: ≥ 3.3	Follow-up mortality: 540 (44.73%)
		Survived	667	363 (54.42)	Mean ± SD: 63.3 ± 13		Mean ± SD: 2.16 ± 1.04 Median (IQR): 2.1 (1.5–2.9)	
		Death	540	326 (60.37)	Mean ± SD: 72.2 ± 9.9		Mean ± SD: 2.8 ± 1.41 Median (IQR): 2.7 (1.9–3.8)	
Tamaki et al. 2023 [61]	Cross-sectional	Total	1026	462 (45.03)	Mean ± SD: 82.33 ± 7.42 Median (IQR): 83 (77-87)	Median: 429 days	Mean ± SD: 4.26 ± 2.74 Median (IQR): 3.9 (2.6–6.3) NLR cut-off: 4.50	Follow-up mortality: 195 (19%)
Liu et al. 2023 [62]	Cross-sectional	Total	1169	673 (57.57)	Mean ± SD: 69.51 ± 13.83	NA	Mean ± SD: 8.43 ± 6.21 Median (IQR): 7.46 (4.73–13.10) NLR tertiles: T1: < 5.43 T2: 5.43–10.33 T3: ≥ 10.33	In-hospital mortality: 183 (15.65%)
		Survived	986	556 (56.39)	Mean ± SD: 68.87 ± 13.96		Mean ± SD: 7.82 ± 5.44 Median (IQR): 7.11 (4.52–11.85)	
		Death	183	117 (63.93)	Mean ± SD: 72.95 ± 12.64		Mean ± SD: 13.43 ± 12.58 Median (IQR): 10.93 (6.26–23.10)	
Zhu et al. 2022 [63]	Prospective cohort	Total	538	357 (66.36)	Mean ± SD: 61.07 ± 15.98	Median: 34 months	Mean ± SD: 2.97 ± 1.96 Median (IQR): 2.64 (1.82–4.47) NLR cut-off: 2.28	Follow-up mortality: 227 (42.19%)
		Survived	311	228 (73.31)	Mean ± SD: 58.03 ± 15.91		Mean ± SD: 2.69 ± 1.78 Median (IQR): 2.40 (1.64–4.04)	
		Death	227	129 (56.83)	Mean ± SD: 65.23 ± 15.16		Mean ± SD: 3.46 ± 2.29 Median (IQR): 3.05 (2.14–5.21)	
Wang et al. 2022 [64]	Cross-sectional	Total	189	106 (56.08)	Mean ± SD: 67.07 ± 13.41	NR	Mean ± SD: 3.46 ± 2.62 NLR cut-off: 2.15	HF detection
Maeda et al. 2022 [65]	Cross-sectional	Total	669	398 (59.49)	Mean ± SD: 75.8 ± 11.3	Median (IQR): 476 (147–796) days	Mean ± SD: 2.62 ± 1.45 Median (IQR): 2.41 (1.75–3.71)	Follow-up mortality & rehospitalization: 255 (38.11%)
Liu et al. 2022 [25]	Retrospective cohort	Total	454	247 (54.41)	Mean ± SD: 76 ± 8	18 months	Mean ± SD: 2.74 ± 1.36 Median (IQR): 2.62 (1.89–3.72) NLR cut-off: 2.53	Follow-up mortality: 42 (9.25%) Rehospitalization: 221 (48.67%)
		Positive major cardiac events	236	131 (55.51)	Mean ± SD: 77 ± 8		Mean ± SD: 3.17 ± 1.53 Median (IQR): 3.03 (2.21–4.27)	
		Negative major cardiac events	218	116 (53.21)	Mean ± SD: 75 ± 7		Mean ± SD: 2.31 ± 1.07 Median (IQR): 2.27 (1.61–3.05)	
Li et al. 2022 [66]	Cross-sectional	Total	50	30 (60.00)	Mean ± SD: 74.16 ± 2.94	6 months	Mean ± SD: 3.50 ± 1.78 NLR cut-off: 3.96	Major cardiac events: 13 (26%)
		Positive major cardiac events	13	8 (61.54)	Mean ± SD: 75.77 ± 3.54		Mean ± SD: 5.12 ± 2.81	
		Negative major cardiac events	37	22 (59.46)	Mean ± SD: 73.59 ± 2.51		Mean ± SD: 2.93 ± 0.64	
Kocaoglu et al. 2022 [67]	Cross-sectional	Total	101	49 (48.51)	Mean ± SD: 73.15 ± 10.19	3 months	Mean ± SD: 6.74 ± 5.44 NLR cut-off: 8.4	Follow-up mortality: 39 (38.61%)
		Survived	62	30 (48.39)	Mean ± SD: 72.61 ± 10.92		Mean ± SD: 5.54 ± 2.98 Median (IQR): 4.95 (3.88–7.81)	
		Death	39	19 (48.72)	Mean ± SD: 74.00 ± 8.96		Mean ± SD: 8.66 ± 7.59 Median (IQR): 6.67 (4.73–14.6)	
Davison et al. 2022 [68]	Cross-sectional	Total	1823	1116 (61.22)	Mean ± SD: 71.40 ± 10.53	6 months	NLR tertiles: T1: ≤ 3.26 T2: 3.26–5.17 T3: ≥ 5.17	Follow-up mortality: 183 (10.03%)
Davran et al. 2022 [22]	Cross-sectional	Total	139	64 (46.04)	Mean ± SD: 69.2 ± 12.1	1 year	Mean ± SD: 6.31 ± 4.48	Follow-up mortality: 14 (10.07%) In-hospital mortality: 9 (6.47%)
		Survived	116	57 (49.14)	Mean ± SD: 69.3 ± 11.5		Mean ± SD: 5.84 ± 4.05	
		Death	23	7 (30.43)	Mean ± SD: 69.2 ± 15		Mean ± SD: 8.7 ± 5.79	
Delcea et al. 2021 [23]	Retrospective cohort	Total	1299	624 (48.04)	Mean ± SD: 72.35 ± 10.45	NA	Mean ± SD: 3.18 ± 1.72 Median (IQR): 2.97 (2.12–4.45) NLR tertiles: T1: 0.89–2.38 T2: 2.39–3.68 T3: 3.69–26.11 NLR cut-off: 3.68	In-hospital mortality: 37 (2.84%) Extended length of hospital stay: 288 (22.17%)
Curran et al. 2021 [34]	Cross-sectional	Total	1622	1086 (66.95)	Mean ± SD: 74 ± 10	Median: 18 months	Median: 3.22	Follow-up mortality: 447 (27.55%) Rehospitalization: 406 (25.03%)
Bai et al. 2021 [35]	Cross-sectional	Total	172	89 (51.74)	Mean ± SD: 71.1 ± 12.5	NA	Mean ± SD: 3.98 ± 2.48 Median (IQR): 3.77 (2.43–5.76)	Heart failure detection
Arfsten et al. 2021 [36]	Cross-sectional	Total	443	325 (73.36)	Mean ± SD: 63 ± 14.13 Median (IQR): 64 (53-72)	Median (IQR): 21 (10-28) months	Mean ± SD: 4.03 ± 2.3 Median (IQR): 3.8 (2.6–5.7)	Follow-up mortality: 75 (16.93%)
Angkananard et al. 2021 [37]	Retrospective cohort	Total	321	144 (44.86)	Mean ± SD: 67.4 ± 14.9	Median (IQR): 23 (2-33) months	Mean ± SD: 3.7 ± 2.45 Median (IQR): 3.2 (2.3–5.6) NLR cut-off: In-hospital mortality: 3.29 Rehospitalization: 3.58 Cardiovascular event: 3.29 Composite outcome: 3.32	Follow-up mortality: 106 (33.02%) In-hospital mortality: 21 (6.54%) Rehospitalization: 62 (19.31%)
Urbanowicz et al. 2020 [38]	Cross-sectional	Total	41	36 (87.80)	Mean ± SD: 50 ± 10	NA	Mean ± SD: 3.46 ± 1.69 Median (IQR): 3.2 (2.5–4.7)	Pulmonary vascular resistance Right ventricular systolic pressure
Sadeghi et al. 2020 [33]	Cross-sectional	Total	197	121 (61.42)	Mean ± SD: 66.31 ± 14.9	6 months	Mean ± SD: 4.41 ± 3.64 NLR cut-off: 7.50	Follow-up mortality: 30 (15.22%)
		Survived	167	110 (65.87)	Mean ± SD: 65.9 ± 14.64		Mean ± SD: 3.84 ± 2.82	
		Death	30	11 (36.67)	Mean ± SD: 68.63 ± 16.82		Mean ± SD: 7.61 ± 5.62	
Kose et al. 2020 [39]	Retrospective cohort	Total	200	146 (73.00)	Mean ± SD: 65 ± 13.6	Mean: 12 months	Mean ± SD: 4.25 ± 3.52 NLR cut-off: 3.70	Follow-up mortality: 38 (19%)
		Survived	162	116 (71.60)	Mean ± SD: 64.6 ± 13.5		Mean ± SD: 3.84 ± 3.28	
		Death	38	30 (78.95)	Mean ± SD: 66.6 ± 13.73		Mean ± SD: 5.98 ± 4.01	
Cho et al. 2020 [40]	Retrospective cohort	Total	5580	2964 (53.12)	Mean ± SD: 68.47 ± 14.4	Mean: 3 years	NLR quartiles: Q1: 0.2–2.0 Q2: 2.1–3.2 Q3: 3.3–5.8 Q4: 5.9–192.4 NLR cut-off: Positive infection and/or ischemia: 7.0 Negative infection and/or ischemia: 5.0	Follow-up mortality: 1891/5301 (35.67%) In-hospital mortality: 331 (5.93%)
Turcato et al. 2019 [41]	Cross-sectional	Total	439	247 (56.26)	Mean ± SD: 81.51 ± 8.2	30 days	Mean ± SD: 5.48 ± 6.61 NLR cut-off: 5.70	Follow-up mortality: 45 (10.25%)
		Survived	394	221 (56.09)	Mean ± SD: 81 ± 8.18 Median (IQR): 82 (75-86)		Mean ± SD: 4.43 ± 2.97 Median (IQR): 4.1 (2.6–6.6)	
		Death	45	26 (57.78)	Mean ± SD: 86 ± 7.65 Median (IQR): 86 (81-91)		Mean ± SD: 14.76 ± 16.08 Median (IQR): 11.7 (5.8–26.8)	
Kone et al. 2019 [42]	Cross-sectional	Total	105	68 (64.76)	Mean ± SD: 63.18 ± 12.8	NA	Mean ± SD: 2.64 ± 1.9	Severe HF prediction
		Moderate HF	81	56 (69.14)	Mean ± SD: 64.3 ± 12.9		Mean ± SD: 2.32 ± 1.07	
		Severe HF	24	12 (50.00)	Mean ± SD: 59.43 ± 12		Mean ± SD: 3.75 ± 3.29	
Boralkar et al. 2019 [43]	Cross-sectional	Total	443	184 (41.53)	Mean ± SD: 76.7 ± 15.5	Median (IQR): 2.2 (0.3–4.9) years	Mean ± SD: 7.06 ± 5.57 Median (IQR): 6.5 (3.6–11.1)	Follow-up mortality: 121 (27.31%)
Yurtdas et al. 2018 [44]	Cross-sectional	Total	40	19 (47.50)	Mean ± SD: 69 ± 12	NA	Mean ± SD: 3.2 ± 1.4	HF detection
Yan et al. 2017 & 2016 [45, 46]	Cross-sectional	Total	1355	816 (60.22)	Mean ± SD: 72.6 ± 8	Median (IQR): 18 (12-29) months	Mean ± SD: 3.2 ± 3.1 NLR tertiles: T1: < 1.96 T2: 1.96–2.90 T3: > 2.90	Follow-up mortality: 92 (6.78%) Rehospitalization: 334 (24.64%) Atrial fibrillation prediction Chronic kidney disease prediction
		Positive major cardiac events	422	280 (66.35)	Mean ± SD: 73.9 ± 8.2		Mean ± SD: 3.6 ± 3.1	
		Negative major cardiac events	933	536 (57.45)	Mean ± SD: 71.9 ± 7.8		Mean ± SD: 3 ± 3	
Pourafkari et al. 2017 [24]	Cross-sectional	Total (In-hospital)	554	531 (95.85)	Mean ± SD: 76.47 ± 11.6	NA	Mean ± SD: 6.3 ± 4.99	In-hospital Mortality: 31 (5.59%)
		Survived	523	500 (95.60)	Mean ± SD: 76 ± 11.6		Mean ± SD: 6.2 ± 4.8	
		Death	31	31 (100.00)	Mean ± SD: 84.5 ± 8.8		Mean ± SD: 8 ± 7.5	
		Total (long-term)	333	319 (95.80)	Mean ± SD: 76.64 ± 11.4	NR	Mean ± SD: 6.13 ± 4.51	Follow-up mortality: 198 (59.45%)
		Survived	135	127 (94.07)	Mean ± SD: 72.9 ± 11		Mean ± SD: 5.6 ± 4.2	
		Death	198	192 (96.97)	Mean ± SD: 79.2 ± 11.1		Mean ± SD: 6.5 ± 4.7	
Huang et al. 2017 [47]	Cross-sectional	Total	1923	1307 (67.97)	Mean ± SD: 76 ± 12	Mean ± SD: 28.6 ± 20.7 months	Mean ± SD: 5.44 ± 6.09	Follow-up mortality: 875 (45.50%)
		Survived	1048	697 (66.51)	Mean ± SD: 74.8 ± 13.9		Mean ± SD: 4.76 ± 5.35	
		Death	875	610 (69.71)	Mean ± SD: 78.3 ± 10.7		Mean ± SD: 6.26 ± 6.8	
Siniorakis et al. 2017 [48]	Cross-sectional	Total	72	31 (43.06)	Mean ± SD: 77 ± 10	NA	Mean ± SD: 3.13 ± 2.38 NLR cut-off: 3.15	In-hospital mortality: 3 (4%) HF differentiation from respiratory infection
Wasilewski et al. 2016 [49]	Cross-sectional	Total	1734	1387 (79.99)	Mean ± SD: 61.66 ± 13.3 Median (IQR): 61 (53-71)	Median (IQR): 660 (331–1074) days	Mean ± SD: 2.93 ± 1.81 NLR tertiles: T1: ≤ 2.04 T2: 2.05–3.1 T3: > 3.1	Follow-up mortality: 443 (25.54%)
Liu et al. 2016 [50]	Cross-sectional	Total	179	96 (53.63)	Mean ± SD: 67.48 ± 13.1	NA	Mean ± SD: 4.26 ± 5.45 NLR cut-off: In-hospital mortality: 3.31 Severe HF: 2.18	In-hospital mortality: 10 (5.58%)
		Survived	169	92 (54.44)	Mean ± SD: 67.1 ± 13		Mean ± SD: 3.9 ± 5.2	
		Death	10	4 (40.00)	Mean ± SD: 74.2 ± 10.5		Mean ± SD: 10.2 ± 6.2	
Argan et al. 2016 [51]	Cross-sectional	Total	68	37 (54.41)	Mean ± SD: 61.33 ± 12.8 Median (IQR): 61 (53-70)	Mean (range): 16 (1-39) months	Mean ± SD: 2.64 ± 1.33 Median (IQR): 2.56 (1.8–3.56) NLR cut-off: 3.0	Progression to kidney disease: 17/48 (35.41%) All cause death and hospitalization: 32/63 (50.79%)
Fu et al. 2015 [52]	Cross-sectional	Total	306	248 (81.05)	Mean ± SD: 84.66 ± 6.7 Median (IQR): 85 (80-89)	Mean: 471 days	Mean ± SD: 3.3 ± 2.23 Median (IQR): 2.9 (2-5)	Follow-up mortality: 104 (33.98%)
Durmus et al. 2015 [53]	Cross-sectional	Total	56	32 (57.14)	Mean ± SD: 67.5 ± 12.6	Mean ± SD: 12.8 ± 7.6 months	Mean ± SD: 5.5 ± 2.8 NLR cut-off: Mortality: 5.1 HF prediction: 3.0	Follow-up mortality: 10 (17.85%)
Cakici et al. 2014 [54]	Cross-sectional	Total	94	59 (62.77)	Mean ± SD: 56.7 ± 10.9	NA	Mean ± SD: 3.33 ± 3.91 Median (IQR): 2.6 (1.1–6.3) NLR cut-off: 2.74	Poor functional capacity
Budak et al. 2014 [55]	Cross-sectional	Total	190	102 (53.68)	Mean ± SD: 68.25 ± 7.8 Median (range): 71 (42-89)	NA	Mean ± SD: 6.2 ± 8.6	NR
Benites-Zapata et al. 2014 [56]	Cross-sectional	Total	527	383 (72.68)	Mean ± SD: 55.43 ± 12.2	Median (IQR): 11.3 (3.4–21.1) months	Mean ± SD: 4.3 ± 2.97 Median (IQR): 3.9 (2.5–6.5) NLR tertiles: T1: < 3 T2: 3.5–5.4 T3: > 5.4	Mortality and heart transplantation: 263 (49.90%)
Turfan et al. 2013 [57]	Cross-sectional	Total	167	101 (60.48)	Mean ± SD: 67.71 ± 9.1	NA	Mean ± SD: 5.01 ± 3.25 NLR cut-off: 4.78	In-hospital mortality: 15 (8.98%)
		Survived	152	95 (62.50)	Mean ± SD: 67 ± 9		Mean ± SD: 4.83 ± 3	
		Death	15	6 (40.00)	Mean ± SD: 75 ± 8		Mean ± SD: 7.2 ± 4.8	
Tasal et al. 2013 [58]	Cross-sectional	Total	219	168 (76.71)	Mean ± SD: 63.2 ± 12.7	NA	Mean ± SD: 6.94 ± 6.26 NLR cut-off: 5.54	In-hospital mortality: 45 (20.54%)
		Survived	174	137 (78.74)	Mean ± SD: 62.5 ± 12.9		Mean ± SD: 6.1 ± 5.3	
		Death	45	31 (68.89)	Mean ± SD: 65.4 ± 11.2		Mean ± SD: 10.2 ± 8.4	
Uthamalingam et al. 2010 [59]	Cross-sectional	Total	1212	606 (50.00)	Mean ± SD: 73.99 ± 13.5	Median (IQR): 26 (15-36) months	Mean ± SD: 6.06 ± 3.93	Follow-up mortality: 284 (23.43%) In-hospital mortality: 63 (5.19%)

*NLR* neutrophil to lymphocyte ratio, *SD* standard deviation, *IQR* interquartile range, *HF* heart failure, *Q* quartile, *T* tertile, *NA* not applicable, *NR* not reported

**Fig. 3:**
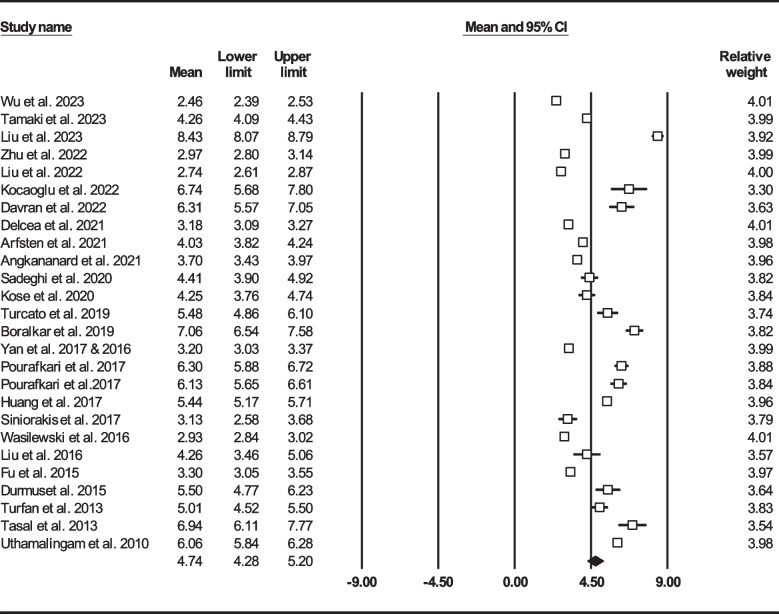
Forest plot for mean NLR based on studies reported mortality (follow-up or in-hospital mortality)

**Fig. 4:**
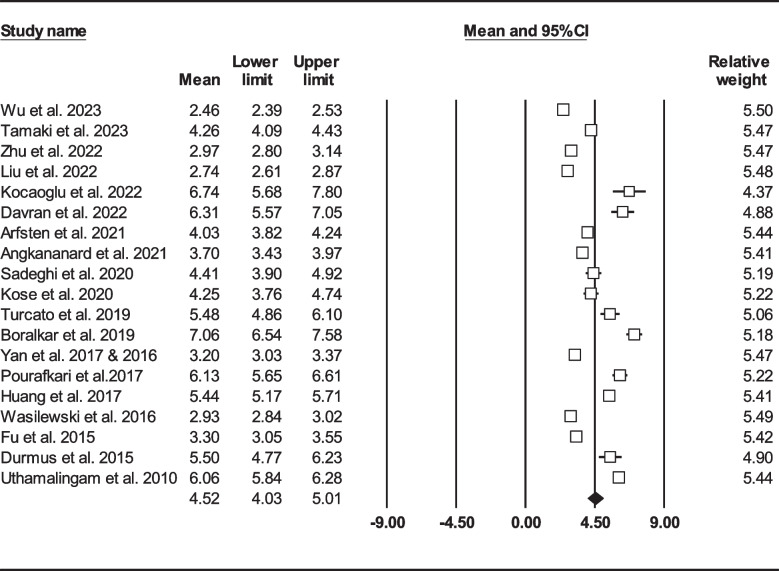
Forest plot for mean NLR based on studies reported follow-up mortality

**Fig. 5:**
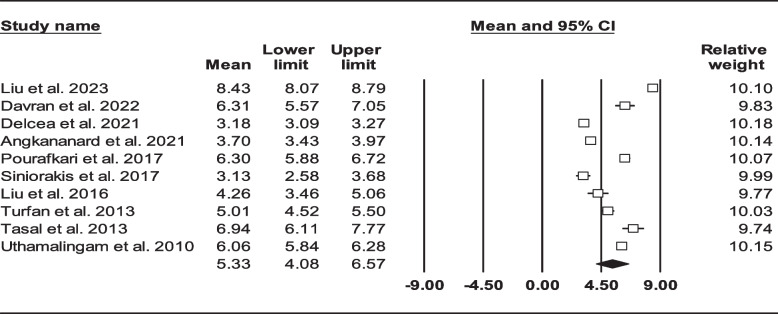
Forest plot for mean NLR based on studies reported in-hospital mortality

We found 25 studies reported total mortality (either follow-up or in-hospital death) on 16086 HF sufferers [22–25, 33, 36, 39, 41, 43, 45–50, 52, 53, 57–63, 67]. Total mean age was 71.62 ± 13.51 years (males: 62%) and 3895 (24.21%) patients died either during admission or follow-up. Figure 3 shows forest plot for mean NLR (4.74, 95% CI: 4.28–5.20). In 19 records (*n* = 12,427, age: 71.75 ± 13.80, males: 62.36%), follow-up mortality had been reported [22, 24, 25, 33, 36, 37, 39, 41, 43, 45–47, 49, 52, 53, 59–61, 63, 67]. Mean NLR in HF subjects was determined to be 4.52 (95% CI: 4.03–5.01) (Fig. 4). On the other hand, we found 10 records (*n* = 5331) reported mortality during hospitalization (age: 71.52 ± 12.94, males: 56.98%), with total NLR mean of 5.33 (95% CI: 4.08–6.57) (Fig. 5) [22–24, 37, 48, 50, 57–59, 62].

**Fig. 6:**
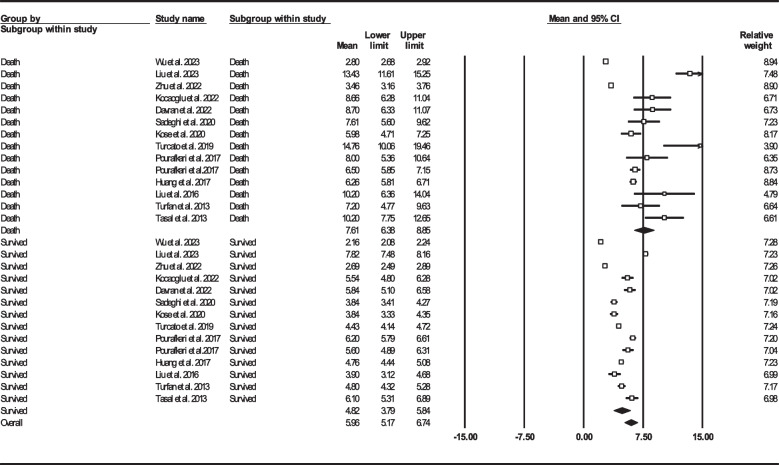
Forest plot for mean NLR based on studies reported death and survived groups

**Fig. 7:**
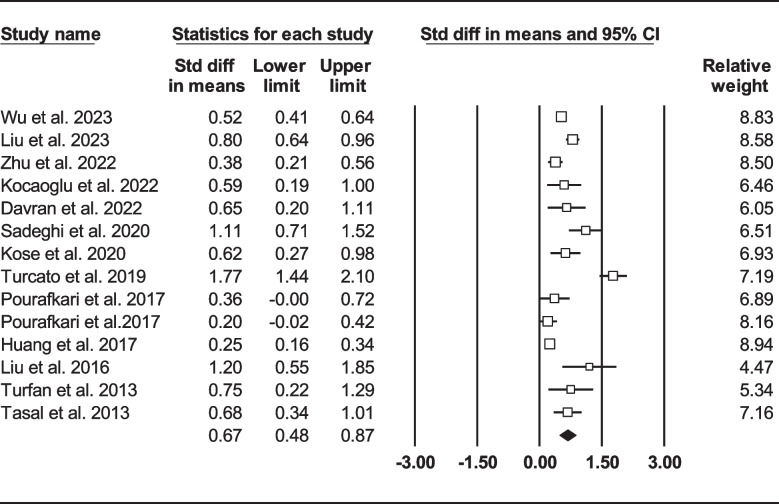
Forest plot for NLR standard mean difference among dead subjects compared to survived ones

**Fig. 8:**
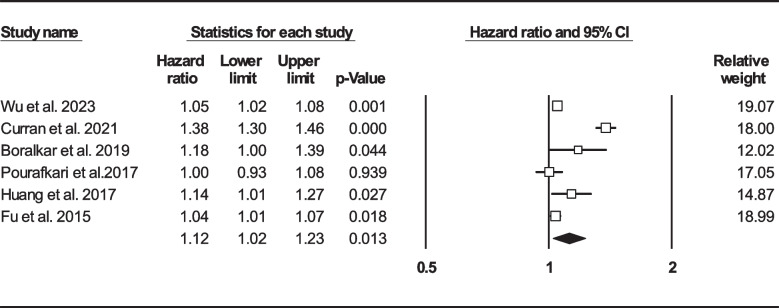
Forest plot for NLR (as continuous variable) mortality hazard ratio

**Fig. 9:**
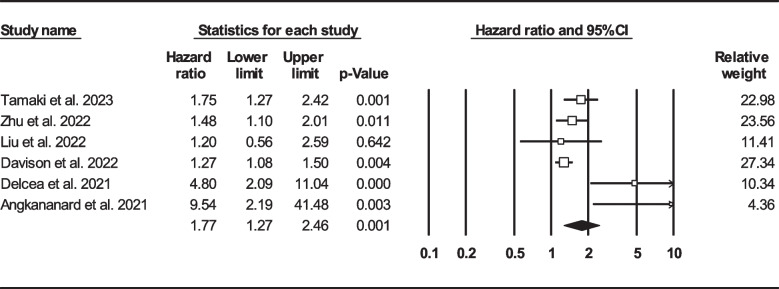
Forest plot for NLR (as dichotomous variable) mortality hazard ratio

**Fig. 10:**
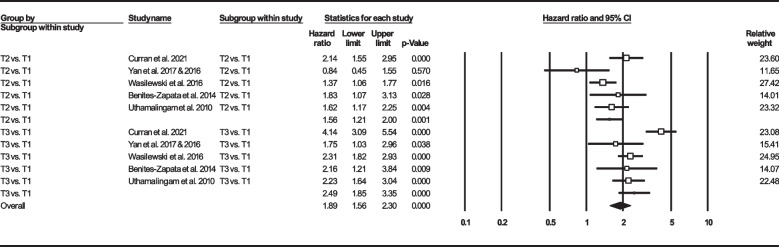
Forest plot for NLR mortality hazard ratio based on NLR tertiles

Thirteen records were selected reporting NLR in dead as well as survived HF subjects [22, 24, 33, 39, 41, 47, 50, 57, 58, 60, 62, 63, 67]. Of 7365 patients, 2299 (31.21%) died. Deceased patients had significantly higher NLR values (7.61, 95% CI: 6.38–8.85) than survivors (4.82, 95% CI: 3.79–5.84) (Fig. 6). Forest plot (Fig. 7) also showed a statistically significant difference in NLR between dead and survived individuals (standardized mean difference: 0.67, 95% CI: 0.48–0.87, *P* < 0.001). Fourteen records (NLR as continuous variable in seven studies, NLR as dichotomous variable in the remaining ones) reported all-cause mortality through multi-variated adjusted HR models based on NLR [23–25, 33, 34, 36, 37, 43, 47, 52, 60, 61, 63, 68]. Due to inconsistent HR and 95% CI in one record in each group, six studies reported NLR as a continuous variable and six studies reported as a dichotomous variable were finally selected. Increasing NLR was associated with higher hazard of death (continuous variable: HR: 1.12, 95% CI: 1.02–1.23, *P* = 0.013 (Fig. 8); dichotomous variable: HR: 1.77, 95% CI: 1.27–2.46, *P* = 0.001) (Fig. 9). We also analyzed mortality based on NLR tertiles (5 out of 6 studies due to inconsistent CIs). Patients in higher NLR tertiles had higher mortality risk than those in the lowest tertile (T2 vs. T1: HR: 1.56, 95% CI: 1.21–2.00, *P* = 0.001; T3 vs. T1: HR: 2.49, 95% CI: 1.85–3.35, *P* < 0.001) (Fig. 10).

**Fig. 11:**
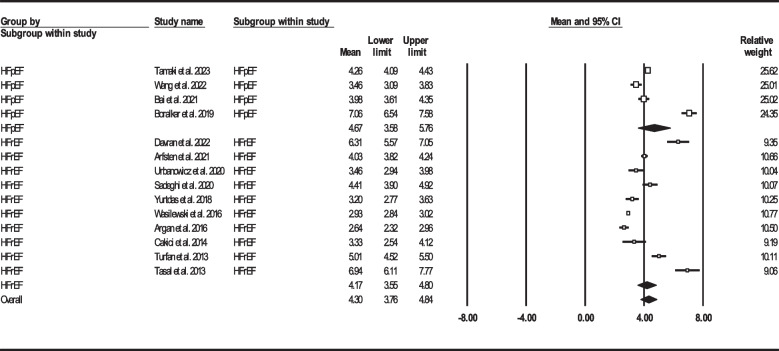
Forest plot for mean NLR based on heart failure status. HFpEF: Heart failure with preserved ejection fraction, HFrEF: Heart failure with reduced ejection fraction

In order to perform subgroup analysis to investigate the probable NLR difference between HF with preserved ejection fraction (HFpEF) and HF with reduced ejection fraction (HFrEF), 14 articles were reported this biomarker among HF sufferers with either preserved (*N* = 4) or reduced (*N* = 10) ejection fraction [22, 33, 35, 36, 38, 43, 44, 49, 51, 54, 57, 58, 61, 64]. Figure 11 shows the forest plot for mean NLR in HFpEF and HFrEF groups. The results failed to prove any significant difference (NLR: 4.67, 95% CI: 3.58–5.76 vs. NLR: 4.17, 95% CI: 3.55–4.80, respectively).

Six articles reported readmission and/or death in HF individuals based on NLR [25, 34, 37, 45, 65, 66]. In Maeda et al.’s study on 669 HF subjects, 255 patients experienced death or HF readmission during the median follow-up of 476 days [65]. In another study, patients with NLR > 2.53 had higher risk of HF rehospitalization (HR: 1.75, 95% CI: 1.26–2.42, *P* = 0.001) but not cardiac death (HR: 1.20, 95% CI: 0.56–2.61, *P* = 0.640) [25]. Li et al. found that HF patients with higher NLR values had higher odds of the primary endpoint (death, HF readmission, or non-fatal myocardial infarction) than those with lower NLR values (odds ratio (OR): 1.631, 95% CI: 1.182–2.248, *P* = 0.019) [66]. Curran and colleagues followed 1622 HF patients for a median of 18 months. During this time, 406 (25.03%) hospitalizations and 447 (27.55%) deaths were reported. A multivariable-adjusted HR model revealed that each SD increase in NLR was associated with a 1.18-fold increase in the risk of mortality and/or rehospitalization (HR: 1.18, 95% CI: 1.11–1.26, *P* < 0.001). Further analysis based on NLR tertiles showed similar results (T2 vs. T1: HR: 1.33, 95% CI: 1.06–1.67, *P* = 0.014; T3 vs. T1: HR: 1.72, 95% CI: 1.37–2.15, *P* < 0.001) [34]. Yan et al.’s findings on 1355 old HF individuals revealed 3^rd^ NLR tertile was associated with higher risk of readmission rather than the 1^st^ tertile (HR: 1.461, 95% CI: 1.108–1.927, *P* = 0.007) [46]. Another study on 321 HF patients indicated HF readmission rate was 19.3% after median (IQR) follow-up period of 23 [2–33] months and patients with higher NLR had 2.70 (95% CI: 1.58–4.61, *P* < 0.001) times increased likelihood of readmission [37].

Four cross-sectional articles reported utility of NLR as a tool to predict HF [35, 42, 44, 64]. Wang and colleagues selected 141 HFpEF patients with New York heart association (NYHA) II- IV and 48 ones with NYHA I as controls, and found NLR was an independent HF presence predictor (OR: 1.388, 95% CI: 1.031–1.870, *P* = 0.031) [64]. In another study on 172 HFpEF patients and 173 controls, multi-variable adjusted regression model revealed NLR was independently associated with HFpEF (OR: 2.351, 95% CI: 1.464–3.776, *P* < 0.001) [35]. In contrast, another study on 40 HF with left ventricular ejection fraction (LVEF) < 40% and 30 healthy controls indicated this biomarker was insignificantly associated with odds of HF detection (OR: 0.644, 95% CI: 0.317–1.309, *P* = 0.224) [44]. Kone et al. enrolled 81 and 24 patients with moderate (NYHA I, II) and severe (NYHA III, IV) HF, respectively, and found patients with NLR of more than 3.0 had 6.78 (95% CI: 1.40–32.80, *P* = 0.017) times higher chances of severe HF rather than the lower group [42].

In terms of hospital stay, a study of 1299 HF patients found that 22.1% had a longer hospital stay (defined as more than seven days admission), and NLR > 3.68 was associated with 1.48-fold higher odds of a longer stay (95% CI: 1.05–2.08, *P* = 0.025) [23].

With regard to the right heart characteristics, an observational study reported pulmonary vascular resistance (PVR) (median (IQR)) and right ventricular systolic pressure (RVSP) (median (IQR)) were significantly different between patients with higher NLR values compared to the other group (PVR: NLR > 6: 407 (186–690) dyn*s*cm^−5^ vs. NLR ≤ 6: 142 (99.5–244.3) dyn*s*cm^−5^, *P* = 0.0386), RVSP: NLR > 6: 60 (40-65) mmHg vs. NLR ≤ 6: 40 (32–49), *P* = 0.0438). They concluded this biomarker could be a useful tool to assess HF progression [38]. One record on 1355 HF individuals (mean NLR: 3.2 ± 3.1) suggested this biomarker could be an independent risk factor for AF (OR: 1.079, 95% CI: 1.027–1.134, *P* = 0.003) [45]. In two studies, NLR association with renal disease was investigated. In the first one, NLR was found to be an independent predictor of kidney disease progression (HR: 1.361, 95% CI: 1.102–1.680, *P* = 0.003) among HFrEF patients with AF [51]. Likewise in another study, NLR was determined to be independently associated with chronic kidney disease (OR: 1.170, 95% CI: 1.054–1.298, P = 0.003) [46]. Finally, functional capacity was assessed with NLR status in one study, indicating this biomarker as an independent predictor of poor functional class in HF (OR: 3.085, 95% CI: 1.520–6.260, *P* = 0.002) [54].

Six articles reported specific NLR tertiles and their associations with clinical HF outcomes [23, 46, 49, 56, 62, 68]. Liu and colleagues assessed the association of NLR and in-hospital mortality on 1169 acute HF subjects, and reported 32 (17.49%), 58 (31.69%), and 93 (50.82%) deaths during hospital admission in each NLR tertile. Patients within the highest NLR tertile had significantly increased chance of in-hospital mortality in comparison to the 1^st^ tertile (OR: 1.06, 95% CI: 1.00–1.11, *P* = 0.035) [62]. In Davison et al.’s study on 1823 acute HF patients, NLR was suggested as an independent predictor of one- and six-month all-cause mortality (HR: 1.66, 95% CI: 1.22–2.25, *P* = 0.001 and HR: 1.27, 95% CI: 1.08–1.50, *P* = 0.003, respectively) [68]. Delcea and colleagues found patients within the 3^rd^ tertile died more frequently during admission (T1: 2 (0.5%), T2: 7 (1.6%) and T3: 28 (6.4%), *P* < 0.001) [23]. Yan et al. stated that HF patients in the highest NLR tertile had a higher risk of major cardiac events (MCE) (composite of cardiac death and HF rehospitalization) during the median follow-up of 18 months (HR: 1.425, 95% CI: 1.109–1.832, *P* = 0.006) [46]. Wasilewski et al. indicated 2^nd^ and 3^rd^ NLR tertiles had been associated with increased hazard of long-term mortality (T2 vs. T1: HR: 1.37, 95% CI: 1.06–1.77, *P* = 0.014, T3 vs. T1: HR: 2.31, 95% CI: 1.82–2.92, *P* < 0.0001, respectively) after follow-up for a median (IQR) of 660 (331–1074) days [49]. Similarly, another study proved patients within the 2^nd^ and 3^rd^ NLR tertiles had 1.61 (95% CI: 1.01–2.37, *P* = 0.02) and 1.55 (95% CI: 1.02–2.36, *P* = 0.04) times increased risk of experiencing primary outcome, defined as death and/or heart transplantation [56].

Two records reported different NLR quartiles and their associations with mortality [40, 60]. Wu et al. found that patients in the highest NLR quartile had a higher risk of mortality than those in the lowest quartile during a median follow-up of 66 months (HR: 1.59, 95% CI: 1.18–2.15, *P* = 0.002) [60]. Another study of 5580 acute HF patients found that those in the highest NLR quartile had significantly higher odds of in-hospital death (OR 2.23, 95% CI: 1.44–3.44, *P* < 0.001) and mortality after three-year follow-up (Q3 vs. Q1: OR: 1.35, 95% CI: 1.16–1.55, *P* < 0.001; Q4 vs. Q1: OR: 1.44, 95% CI: 1.24–1.67, *P* < 0.001) [40].

Nineteen articles reported specific NLR cut-off points and evaluated their relations with different clinical outcomes including the followings: in-hospital or long-term mortality, extended length of hospital stay, HF prediction, rehospitalization, renal disease progression, acute HF differentiation from respiratory infections, poor functional capacity, cardiovascular events plus its composite with all-cause mortality, and prediction of cardiovascular outcomes, defined as cardiac death, non-fatal myocardial infarction, and HF rehospitalization [23, 25, 33, 37, 39–41, 48, 50, 51, 53, 54, 57, 58, 61, 63, 64, 66, 67]. Detailed information of each cut-off value is represented in Table 2.

**Table 2:** Summary of neutrophil to lymphocyte ratio (NLR) cut-off characteristics

Authors	Outcome	NLR cut-off	Sensitivity	Specificity	Area under curve	95% confidence interval	*P*-value
Tamaki et al. 2023 [61]	Follow-up mortality	4.50	NR	NR	NR	NR	NR
Zhu et al. 2022 [63]	Follow-up mortality	2.28	75.1%	48%	0.637	0.584–0.690	NR
Wang et al. 2022 [64]	HF diagnosis	2.15	78.72%	68.09%	0.753	0.685–0.813	NR
Liu et al. 2022 [25]	Follow-up mortality, HF rehospitalization	2.53	NR	NR	NR	NR	NR
Li et al. 2022 [66]	Major adverse cardiac events	3.96	76.92%	100%	0.841	0.678–1.00	< 0.001
Kocaoglu et al. 2022 [67]	Follow-up mortality	8.40	46.15%	79.03%	0.643	NR	0.013
Delcea et al. 2021 [23]	In-hospital mortality	3.68	78.38%	67.20%	0.765	0.693–0.837	< 0.001
	Extended length of hospital stay	3.68	52.61%	71.81%	0.681	0.644–0.717	< 0.001
Angkananard et al. 2021 [37]	In-hospital mortality	3.29	87.5%	70.8%	0.79	0.66–0.91	NR
	Rehospitalization	3.58	61.2%	61%	0.56	0.48–0.64	NR
	Cardiovascular events	3.29	75.2%	66.1%	0.67	0.61–0.72	NR
	Cardiovascular events plus all-cause mortality	3.32	71.6%	86.8%	0.80	0.75–0.85	NR
Sadeghi et al. 2020 [33]	Follow-up mortality	7.50	50%	91.7%	0.708	NR	< 0.001
Kose et al. 2020 [39]	Follow-up mortality	3.70	71.1%	65.6%	0.705	NR	< 0.001
Cho et al. 2020 [40]	In-hospital and follow-up mortality	Positive infection and/or ischemia: 7.0 Negative infection and/or ischemia: 5.0	NR	NR	NR	NR	NR
Turcato et al. 2019 [41]	Follow-up mortality	5.70	NR	NR	0.76	NR	NR
Siniorakis et al. 2017 [48]	HF differentiation from respiratory infection	3.15	82.1%	77.8%	0.773	NR	< 0.001
Liu et al. 2016 [50]	In-hospital mortality	3.31	100%	68.1%	0.885	0.799–0.971	NR
	Severe HF prediction	2.18	87.3%	52.4%	0.701	0.628–0.767	NR
Argan et al. 2016 [51]	Progression to kidney disease	3.0	68%	75%	0.72	0.58–0.85	0.001
Durmus et al. 2015 [53]	Follow-up mortality	5.10	75%	62%	0.730	NR	0.045
Cakici et al. 2014 [54]	Poor functional capacity	2.74	79.4%	80%	0.819	0.731–0.908	< 0.001
Turfan et al. 2013 [57]	In-hospital mortality	4.78	66.7%	60.5%	0.687	NR	NR
Tasal et al. 2013 [58]	In-hospital mortality	5.54	67%	66%	0.73	0.65–0.83	< 0.001

*HF* heart failure, *NLR* neutrophil to lymphocyte ratio, *NR* not reported

### Risk of bias assessment

Tables S1 and S2 showed the results of risk of bias assessment. Six manuscripts performed in a cohort format and others had cross-sectional designs. NLR was defined as division of absolute neutrophil counts over absolute lymphocyte counts [22–25, 33–68]. No study had significant risk of bias and we included all of them for the downstream analysis.

### Publication bias and GRADE assessment

Heterogeneity details as well as funnel plot associated with total NLR mean in HF are shown in Table S3 and Figure S1, respectively. Although funnel plot was in favor of asymmetry (Egger’s test (*P* = 0.00001), Begg’s test (*P* = 0.014)), Duval and Tweedie’s trim-and-fill method revealed similar point estimate between observed and adjusted values (4.376, 95% CI: 4.019–4.733), suggesting no probable publication bias.

Heterogeneity information as well as funnel plot among recruited studies reported total mortality (either follow-up or in-hospital death) are shown in Table S3 and Figure S2, respectively (Egger’s test (*P* = 0.00001), and Begg’s test (*P* = 0.026)). However, Duval and Tweedie’s trim-and-fill method showed similar point estimates and intervals between observed and adjusted values (4.739, 95% CI: 4.283–5.195). Further information on publication bias and heterogeneity indices of enrolled records reporting follow-up death are provided in Figure S3 and Table S3, respectively. The results of Egger’s (*P* = 0.00005) and Begg’s (*P* = 0.008) tests were in favor of presence of funnel plot asymmetry. However, Duval and Tweedie’s trim-and-fill method showed no probable publication bias (similar observed and adjusted point estimate: 4.523, 95% CI: 4.034–5.012). Heterogeneity indices showed considerable heterogeneity in enrolled records indicating in-hospital mortality (Table S3). We also provided funnel plot in Figure S4, indicating asymmetry (Egger’s test (*P* = 0.030), Begg’s test (*P* = 0.500)), but no probable publication bias (Duval and Tweedie’s trim-and-fill method observed and adjusted point estimate: 5.327, 95% CI: 4.084–6.570).

Heterogeneity indices for studies reported NLR among dead or survived HF subjects are shown in Table S3, with further provision of the funnel plot in Figure S5. Our findings suggested presence of possible publication bias (observed point estimate: 5.933, 95% CI: 5.265–6.601, adjusted point estimate: 5.115, 95% CI: 4.485–5.744). Certainty of this outcome is shown in Table S4. Funnel plot, Egger’s (*P* = 0.220), and Begg’s (*P* = 0.129) tests indicated symmetry (Figure S6) and no publication bias (similar observed and adjusted point estimate: 1.120, 95% CI: 1.023–1.226) among studies reported this biomarker’s impact, as a continuous variable, on mortality HR. In terms of NLR based on HF status, the heterogeneity indices are provided in Table S3. Finally, we provided the certainty of all aforementioned outcomes in Table S5.

## Discussion

**Fig. 12:**
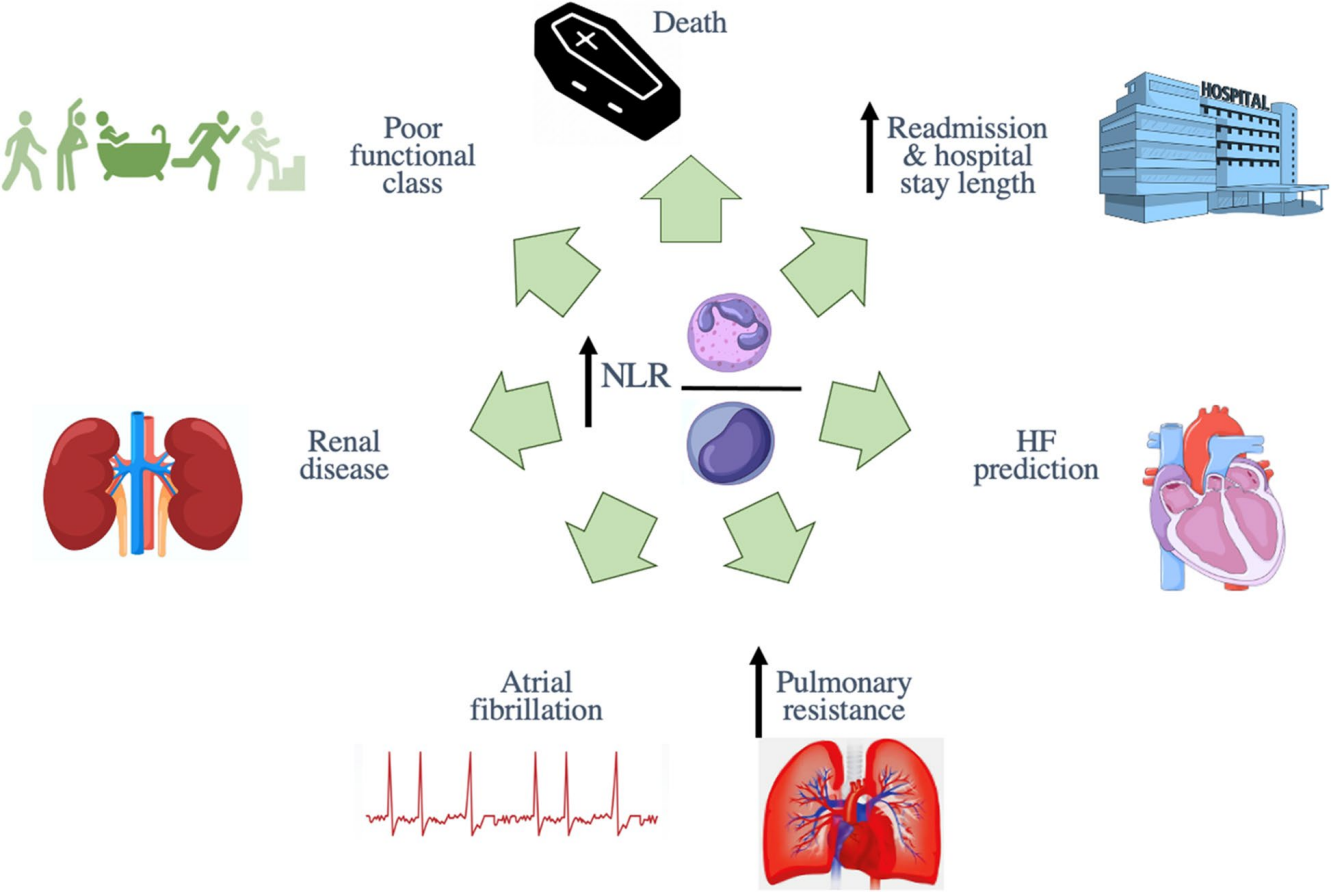
Summary figure of NLR association with different clinical outcomes in HF sufferers

We found that mean NLR in HF patients was 4.38 (95% CI: 4.02–4.73). Each unit increase in this biomarker has been associated with 1.12 (95% CI: 1.02–1.23, *P* = 0.013) times increased mortality risk and this risk was higher among patients with higher NLR values than proposed cut-offs (HR: 1.77, 95% CI: 1.27–2.46, *P* = 0.001). Also, being in a higher NLR tertile had been associated with increased death likelihood (T2 vs. T1: HR: 1.56, 95% CI: 1.21–2.00, *P* = 0.001, T3 vs. T1: HR: 2.49, 95% CI: 1.85–3.35, *P* < 0.001). Furthermore, NLR values were significantly higher in deceased HF subjects compared to survived ones (standard mean difference: 0.67 95% CI: 0.48–0.87, *P* < 0.001). Since this ratio can be easily calculated during admission, it seems NLR could be a useful tool in health care settings for appropriate patients’ risk stratification. Summary figure of NLR association with different clinical outcomes in HF sufferers is provided in Fig. 12.

To date, only one systematic review and meta-analysis was done to assess prognostic utility of NLR in HF. Although the reported mortality HR was significant (HR: 1.28, 95% CI: 1.14–1.43), some points should be noted. They only searched two databases and the literature screening was up to September 2017 and they finally enrolled nine eligible studies to assess all-cause mortality [69].

Although the exact pathophysiological relation between higher NLR and worsening of cardiovascular outcomes has to be elucidated, inflammation is recognized as a main player. It has been previously reported long-term mortality in HF subjects increased as white blood cells increase. Secretion of different inflammatory cytokines including C-reactive protein (CRP), tumor necrosis factor-α (TNF-α) and interleukin (IL)-1 results in reduction in cardiac activity [70–72]. Moreover, neutrophils release multiple proteolytic enzymes like elastase, acid phosphatase and myeloperoxidase leading to destructive effects on cardiac tissue [73, 74]. The secretion of these inflammatory signals, coupled with increased release of granulocyte-monocyte colony-stimulating factor, lipopolysaccharides, hypoxia signals, and free radicals during the inflammatory process, ultimately prolongs the lifespan of neutrophils and induces detrimental effects on the heart [75–77].

On the other hand, lymphocytes play an immunomodulatory action by inducing the expression of tissue inhibitor of metalloproteinase-1 [78]. Activation of hypothalamic–pituitary–adrenal axis in context of HF, as a stressful condition, causes increased cortisol secretion from adrenal glands. This hormone induces lymphocyte apoptosis and consequent lymphocytopenia [19, 78, 79]. Also, TNF-α has been suggested as a culprit in diminishing lymphocyte counts in this regard [80]. In addition to apoptosis, other potential mechanisms proposed to induce lymphocytopenia include neurohormonal activation and downregulation of lymphocyte proliferation and differentiation [81, 82].

NLR has also been implicated in other non-CVDs including irritable bowel syndrome, multiple sclerosis, spontaneous intracerebral hemorrhage, as well as malignancies [83–87]. The strength of NLR as a potential prognostic tool might be attributed to two different immunologic pathways. The first one is associated with neutrophils with a rapid response. On the contrary, lymphocytes modulate a more adaptive and chronic immune system response [15]. Another possible mechanism could be related to NLR association with autonomic nervous system in a way that this ratio could imply sympathetic over parasympathetic autonomic nervous system tone. In case of sympathetic stimulation, granulocyte numbers increase. Conversely, parasympathetic down-stimulation results in decreasing lymphocyte counts leading to higher NLR values [88]. Interestingly, NLR has been suggested to be a better tool rather than its independent components (neutrophils and lymphocytes) for mortality prediction among HF sufferers [56, 59]. Given that complete blood count is routinely conducted for HF patients upon admission and typically provides information about leukocyte subsets, the measurement of NLR for effective risk stratification and the prioritization of high-risk HF patients without the need for additional costly tests presents an interesting prospect for healthcare facilities.

Although data are still limited in association of NLR with AF and renal disease, some possibilities should be considered. In terms of AF, it has been reported that AF occurrence increases with aging, and inflammation has been attributed to AF initiation; thus, co-existence of HF and AF could be predictable among elderly population [89, 90]. Also, any neuro-hormonal and structural alterations in one condition can negatively affect the other disease [91]. For renal disease, despite the fact that the exact mechanism has not been identified yet, the mutual inflammatory cytokines (CRP, IL-1, IL-6 and TNF-α) can invade renal tissue causing interstitial fibrosis, tubular injury and infiltration of different inflammatory cells [51]. Therefore, co-occurrence of HF and chronic renal disease might be associated with worsen clinical outcomes and NLR could be a useful prognostic tool in this regard.

Several strengths could be considered in current study. We tried our best to include all published articles without any time limitations. We also screened four most well-known electronic databases and used a comprehensive search strategy to recruit all potential records.

### Limitations

Current study was not free from limitations. We only enrolled English records and some non-English articles might be missed. There was significant funnel plot asymmetry, probably due to different sample sizes and designs in each study which led to considerable inter-study heterogeneity and possible publication bias. However, other possible sources of this asymmetry, including inadequate analysis, selective analysis or selective outcome reporting should be considered [92]. We were unable to assess NLR difference stratified by gender. Although we implemented HR analysis according to binary variable as well as NLR tertiles, interpretation should be done with cautions due to variable tertile ranges and cut-off points reported in each record. Also, the certainty of evidence ranged from very low to low, most commonly due to considerable heterogeneity among included studies and insufficient number of available studies for most of HF clinical outcomes.

## Conclusions

In conclusion, this systematic review and meta-analysis indicated NLR could be used as a practical prognostic tool for risk stratification and prioritizing high risk patients in the first place during admission and might be used as an independent factor for HF evaluation, especially in resource limited countries. Complementary studies are required clarifying the prognostic capability of NLR.

### Supplementary Information

**Additional file 1.** Supplementary materials.

## Data Availability

The datasets generated during and/or analyzed during the current study are not publicly available due to confidential issues but are available from the corresponding author on reasonable request.

## Abbreviations

AF: Atrial Fibrillation
AXIS: A critical appraisal tool
CI: Confidence Interval
CMA: Comprehensive Meta-Analysis
CRP: C-Reactive Protein
CVDs: CardioVascular Diseases
GRADE: Grading of Recommendations Assessment, Development and Evaluation
HF: Heart Failure
HFpEF: Heart Failure with preserved Ejection Fraction
HFrEF: Heart Failure with reduced Ejection Fraction
HR: Hazard Ratio
IL: Interleukin
IQR: Interquartile Range
JBI: Joanna Briggs Institute
LVEF: Left Ventricular Ejection Fraction
MCE: Major Cardiac Event
MeSH: Medical Subject Headings
NIH: National Institute of Health
NLR: Neutrophil to Lymphocyte Ratio
NYHA: New York Heart Association
OR: Odds Ratio
PECOS: Population, Exposure, Comparator, Outcomes, Study designs
PRISMA: Preferred Reporting Items for Systematic Reviews and Meta-Analyses
PROSPERO: International Prospective Register of Systematic Reviews
PVR: Pulmonary Vascular Resistance
RCT: Randomized Clinical Trial
RVSP: Right Ventricular Systolic Pressure
SD: Standard Deviation
TNF-α: Tumor Necrosis Factor- α
