# Supplementary material for: Neutrophil to lymphocyte ratio (NLR) prognostic effects on heart failure; a systematic review and meta-analysis
Source: BMC Cardiovasc Disord. 2023 Nov 14;23:555. doi: 10.1186/s12872-023-03572-6 (PMC10644447; doi:10.1186/s12872-023-03572-6)
Supplement: Supplementary file 1 — Additional file 1. Supplementary materials. [file 12872_2023_3572_MOESM1_ESM.pdf]

Supplementary materials

Table S1: Risk of bias assessment of cross-sectional studies.

Components	Wu et al. 2023	Tamaki et al. 2023	Liu et al. 2023	Wang et al. 2022	Maeda et al. 2022	Li et al. 2022	Kocaoglu et al. 2022	Davison et al. 2022	Davran et al. 2022	Curran et al. 2021	Bai et al. 2021	Arfsten et al. 2021	Urbanowicz et al. 2020	Sadeghi et al. 2020	Turcato et al. 2019	Kone et al. 2019	Boralkar et al. 2019	Yurtdas et al. 2018	Yan et al. 2017	Pourafkari et al. 2017	Huang et al. 2017	Siniorakis et al. 2017	Yan et al. 2016	Wasilewski et al. 2016	Liu et al. 2016	Argan et al. 2016	Fu et al. 2015	Durmus et al. 2015	Cakici et al. 2014	Budak et al. 2014	Benites-Zapata et al. 2014	Turfan et al. 2013	Tasal et al. 2013	Uthamalingam et al. 2010
1. Were the aims/objectives of the study clear?	Yes	Yes	Yes	Yes	Yes	Yes	Yes	Yes	Yes	Yes	Yes	Yes	Yes	Yes	Yes	Yes	Yes	Yes	Yes	Yes	Yes	Yes	Yes	Yes	Yes	Yes	Yes	Yes	Yes	Yes	Yes	Yes	Yes	Yes

2. Was the study design appropriate for the stated aim(s)?	Yes	Yes	Yes	Yes	Yes	Yes	Yes	Yes	Yes	Yes	Yes	Yes	Yes	Yes	Yes	Yes	Yes	Yes	Yes	Yes	Yes	Yes	Yes	Yes	Yes	Yes	Yes	Yes	Yes	Yes	Yes	Yes	Yes	Yes
3. Was the sample size justified?	Yes	Yes	Yes	Yes	Yes	Yes	Yes	Yes	Yes	Yes	Yes	Yes	Yes	Yes	Yes	Yes	Yes	Yes	Yes	Yes	Yes	Yes	Yes	Yes	Yes	Yes	Yes	Yes	Yes	Yes	Yes	Yes	Yes	Yes

4. Was the target/reference population clearly defined? (Is it clear who the research was about?)	Yes	Yes	Yes	Yes	Yes	Yes	Yes	Yes	Yes	Yes	Yes	Yes	Yes	Yes	Yes	Yes	Yes	Yes	Yes	Yes	Yes	Yes	Yes	Yes	Yes	Yes	Yes	Yes	Yes	Yes	Yes	Yes	Yes	Yes

5. Was the sample frame taken from an appropriate population base so that it closely represented the target/reference population under investigation?	Yes	Yes	Yes	Yes	Yes	Yes	Yes	Yes	Yes	Yes	Yes	Yes	Yes	Yes	Yes	Yes	Yes	Yes	Yes	Yes	Yes	Yes	Yes	Yes	Yes	Yes	Yes	Yes	Yes	Yes	Yes	Yes	Yes	Yes

6. Was the selection process likely to select subjects/participants that were representative of the target/reference population under investigation?	Yes	Yes	Yes	Yes	Yes	Yes	Yes	Yes	Yes	Yes	Yes	Yes	Yes	Yes	Yes	Yes	Yes	Yes	Yes	Yes	Yes	Yes	Yes	Yes	Yes	Yes	Yes	Yes	Yes	Yes	Yes	Yes	Yes	Yes

7. Were measures undertaken to address and categorize non-responders?	Not applicable	Not applicable	Not applicable	Not applicable	Not applicable	Not applicable	Not applicable	Not applicable	Not applicable	Not applicable	Not applicable	Not applicable	Not applicable	Not applicable	Not applicable	Not applicable	Not applicable	Not applicable	Not applicable	Not applicable	Not applicable	Not applicable	Not applicable	Not applicable	Not applicable	Not applicable	Not applicable	Not applicable	Not applicable	Not applicable	Not applicable	Not applicable	Not applicable	Not applicable
8. Were the risk factor and outcome variables measured appropriate to the aims of the study?	Yes	Yes	Yes	Yes	Yes	Yes	Yes	Yes	Yes	Yes	Yes	Yes	Yes	Yes	Yes	Yes	Yes	Yes	Yes	Yes	Yes	Yes	Yes	Yes	Yes	Yes	Yes	Yes	Yes	Yes	Yes	Yes	Yes	Yes

9. Were the risk factor and outcome variables measured correctly using instruments/measurements that had been trialed, piloted or published previously?	Yes	Yes	Yes	Yes	Yes	Yes	Yes	Yes	Yes	Yes	Yes	Yes	Yes	Yes	Yes	Yes	Yes	Yes	Yes	Yes	Yes	Yes	Yes	Yes	Yes	Yes	Yes	Yes	Yes	Yes	Yes	Yes	Yes	Yes

10. Is it clear what was used to deter mined statistical significance and/or precision estimates? (eg, p values, CIs)	Yes	Yes	Yes	Yes	Yes	Yes	Yes	Yes	Yes	Yes	Yes	Yes	Yes	Yes	Yes	Yes	Yes	Yes	Yes	Yes	Yes	Yes	Yes	Yes	Yes	Yes	Yes	Yes	Yes	Yes	Yes	Yes	Yes	Yes

11. Were the methods (including statistical methods) sufficiently described to enable them to be repeated?	Yes	Yes	Yes	Yes	Yes	Yes	Yes	Yes	Yes	Yes	Yes	Yes	Yes	Yes	Yes	Yes	Yes	Yes	Yes	Yes	Yes	Yes	Yes	Yes	Yes	Yes	Yes	Yes	Yes	Yes	Yes	Yes	Yes	Yes
12. Were the basic data adequately described?	Yes	Yes	Yes	Yes	Yes	Yes	Yes	Yes	Yes	Yes	Yes	Yes	Yes	Yes	Yes	Yes	Yes	Yes	Yes	Yes	Yes	Yes	Yes	Yes	Yes	Yes	Yes	Yes	Yes	Yes	Yes	Yes	Yes	Yes

13. Does the response rate raise concerns about non-response bias?	No	No	No	No	No	No	No	No	No	No	No	No	No	No	No	No	No	No	No	No	No	No	No	No	No	No	No	No	No	No	No	No	No	No
14. If appropriate, was information about non-responders described?	Not applicable	Not applicable	Not applicable	Not applicable	Not applicable	Not applicable	Not applicable	Not applicable	Not applicable	Not applicable	Not applicable	Not applicable	Not applicable	Not applicable	Not applicable	Not applicable	Not applicable	Not applicable	Not applicable	Not applicable	Not applicable	Not applicable	Not applicable	Not applicable	Not applicable	Not applicable	Not applicable	Not applicable	Not applicable	Not applicable	Not applicable	Not applicable	Not applicable	Not applicable
15. Were the results internally consistent?	Yes	Yes	Yes	Yes	Yes	Yes	Yes	Yes	Yes	Yes	Yes	Yes	Yes	Yes	Yes	Yes	Yes	Yes	Yes	Yes	Yes	Yes	Yes	Yes	Yes	Yes	Yes	Yes	Yes	Yes	Yes	Yes	Yes	Yes

16. Were the results for the analyses described in the methods, presented?	Yes	Yes	Yes	Yes	Yes	Yes	Yes	Yes	Yes	Yes	Yes	Yes	Yes	Yes	Yes	Yes	Yes	Yes	Yes	Yes	Yes	Yes	Yes	Yes	Yes	Yes	Yes	Yes	Yes	Yes	Yes	Yes	Yes	Yes
17. Were the authors' discussions and conclusions justified by the results?	Yes	Yes	Yes	Yes	Yes	Yes	Yes	Yes	Yes	Yes	Yes	Yes	Yes	Yes	Yes	Yes	Yes	Yes	Yes	Yes	Yes	Yes	Yes	Yes	Yes	Yes	Yes	Yes	Yes	Yes	Yes	Yes	Yes	Yes

18. Were the limitations of the study discussed?	Yes	Yes	Yes	Yes	Yes	No	Yes	Yes	Yes	Yes	Yes	Yes	No	Yes	Yes	Yes	Yes	Yes	Yes	Yes	Yes	No	Yes	Yes	Yes	No	Yes	Yes	Yes	Yes	Yes	Yes	Yes	Yes
19. Were there any funding sources or conflicts of interest that may affect the authors' interpretation of the results?	No	No	No	No	No	No	No	No	No	No	No	No	No	No	No	No	No	No	No	No	No	No	No	No	No	No	No	No	No	No	No	No	No	No

20. Was ethical approval or consent of participants attained?	Yes	Yes	Yes	Yes	Yes	No	Yes	Yes	Yes	Yes	Yes	Yes	No	Yes	Yes	No	No	Yes	Yes	Yes	Yes	Yes	Yes	Yes	Yes	No	Yes	Yes	Yes	Yes	Yes	Yes	Yes	Yes

Table S2: Risk of bias assessment of cohort studies.

Major components	Delcea et al. 2021	Angkananard et al. 2021	Kose et al. 2020	Cho et al. 2020	Zhu et al. 2022	Liu et al. 2022
1. Were the two groups similar and recruited from the same population?	Not applicable	Yes	Yes	Yes	Not applicable	Not applicable
2. Were the exposures measured similarly to assign people to both exposed and unexposed groups?	Not applicable	Not applicable	Yes	Yes	Not applicable	Not applicable
3. Was the exposure measured in a valid and reliable way?	Not applicable	Yes	Yes	Yes	Not applicable	Not applicable
4. Were confounding factors identified?	Yes	Yes	Yes	Yes	Yes	Yes
5. Were strategies to deal with confounding factors stated?	Yes	Yes	Yes	Yes	Yes	Yes
6. Were the groups/participants free of the outcome at the start of the study (or at the moment of exposure)?	Yes	Yes	Yes	Yes	Yes	Yes
7. Were the outcomes measured in a valid and reliable way?	Yes	Yes	Yes	Yes	Yes	Yes
8. Was the follow up time reported and sufficient to be long enough for outcomes to occur?	Yes	Yes	Yes	Yes	Yes	Yes
9. Was follow up complete, and if not, were the reasons to loss to follow up described and explored?	Yes	Yes	Yes	Yes	Yes	Yes
10. Were strategies to address incomplete follow up utilized?	Not applicable	Not applicable	Not applicable	Not applicable	Not applicable	Not applicable
11. Was appropriate statistical analysis used?	Yes	Yes	Yes	Yes	Yes	Yes

Overall appraisal:	Include	Include	Include	Include	Include	Include

Table S3. Heterogeneity results of included studies according to neutrophil to lymphocyte ratio.

Outcome of interest	Q*	I^{2**}	τ^{2***}	P
Mean NLR	3717.161	99.03%	1.167	<0.001
Total mortality	3465.655	99.27%	1.347	<0.001
Follow-up mortality	2285.622	99.21%	1.132	<0.001
In-hospital mortality	1456.922	99.38%	3.947	<0.001
NLR difference (death/survival)	2589.33	98.95%	2.773	<0.001
NLR difference (HFpEF/HFrEF)	658.314	98.02%	1.05	<0.001

*: Cochran's Q statistic for heterogeneity, ** : Index for the degree of heterogeneity, ***: Tau-squared measure of heterogeneity.

NLR: neutrophil to lymphocyte ratio, HFpEF: heart failure with preserved ejection fraction, HFrEF: heart failure with reduced ejection fraction

Table S4. Certainty evidence of NLR difference in deceased compared to survived groups in HF patients.

Certainty assessment							№ of patients		Effect		Certainty	Importance
№ of studies	Study design	Risk of bias	Inconsistency	Indirectness	Imprecision	Other considerations	Deceased	Survived	Relative (95% CI)	Absolute (95% CI)		
**NLR difference**												
13	observational studies	not serious	serious[a]	not serious	not serious	publication bias strongly suspected[b]	2299	5066	-	SMD **0.67 SD more** (0.48 more to 0.87 more)	⊕◯◯◯ Very low	IMPORTANT

CI: confidence interval; **SMD**: standardised mean difference

Explanations

a. Considerable I2 value
b. Evidence of publication bias using Duval and Tweedie's trim-and-fill method

Table S5. Certainty evidence of high NLR compared to low NLR for clinical outcomes in HF subjects.

Certainty assessment							№ of patients		Effect		Certainty	Importance
№ of studies	Study design	Risk of bias	Inconsistency	Indirectness	Imprecision	Other considerations	High NLR	Low NLR	Relative (95% CI)	Absolute (95% CI)		
**Mortality hazard ratio**												
6	observational studies	not serious	serious[a]	not serious	not serious	none			**HR 1.77** (1.27 to 2.46)	**2 fewer per 1,000** (from 2 fewer to 1 fewer)	⊕◯◯◯ Very low	IMPORTANT
**Death and/or re-hospitalization**												
5	observational studies	not serious	not serious	not serious	not serious	none	HF patients with higher NLR values had higher likelihood of death and/or readmission.				⊕⊕◯◯ Low	IMPORTANT
**HF prediction**												
4	observational studies	not serious	not serious	serious[b]	not serious	none	Two studies were in favor of independency of NLR for HF prediction. One study was in contrast to independency of NLR for HF prediction. One study was in favor of independency of NLR for severe HF prediction.				⊕◯◯◯ Very low	IMPORTANT
**Extended length of hospital stay**												
1	observational studies	not serious	not serious	not serious	not serious	none	Increased NLR was associated with higher odds of increased hospital stay length.				⊕⊕◯◯ Low	NOT IMPORTANT
**Pulmonary resistance**												
1	observational studies	not serious	not serious	not serious	not serious	none	HF patients with higher NLR had higher pulmonary vascular resistance and right ventricular systolic pressure compared to patients with lower NLR values.				⊕⊕◯◯ Low	NOT IMPORTANT
**Atrial fibrillation**												
1	observational studies	not serious	not serious	not serious	not serious	none	NLR was an independent predictor of atrial fibrillation in HF patients.				⊕⊕◯◯ Low	NOT IMPORTANT
**Renal disease**												
2	observational studies	not serious	not serious	not serious	not serious	none	All studies reported NLR as an independent factor for renal disease.				⊕⊕◯◯ Low	NOT IMPORTANT
**Functional class**												
1	observational studies	not serious	not serious	not serious	not serious	none	NLR was an independent predictor of poor functional class in HF.				⊕⊕◯◯ Low	NOT IMPORTANT

CI: confidence interval; **HR:** hazard Ratio; **NLR:** neutrophil to lymphocyte ratio; **HF:** heart failure

Explanations

a. Considerable I2 value
b. Studies with different reports.

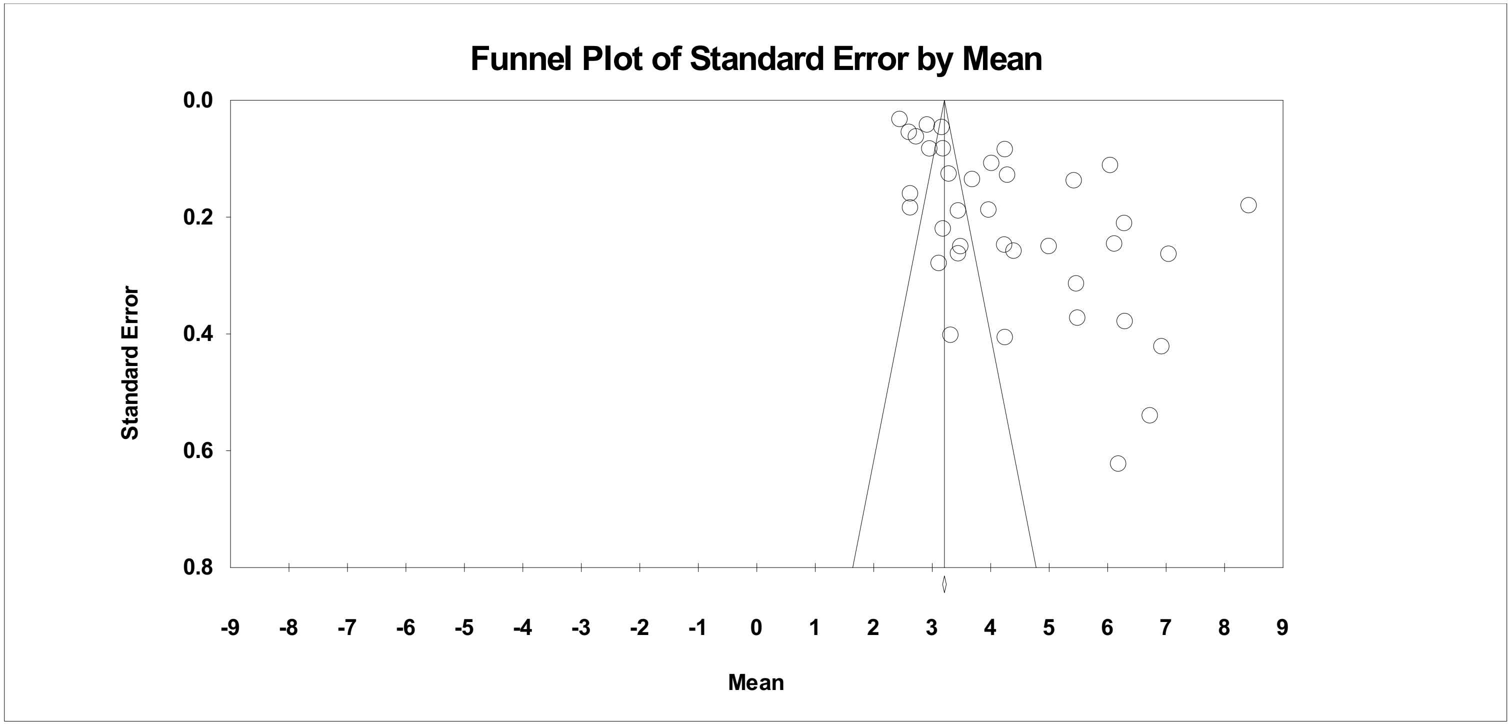

Figure S1. Funnel plot for mean NLR based on total study population.

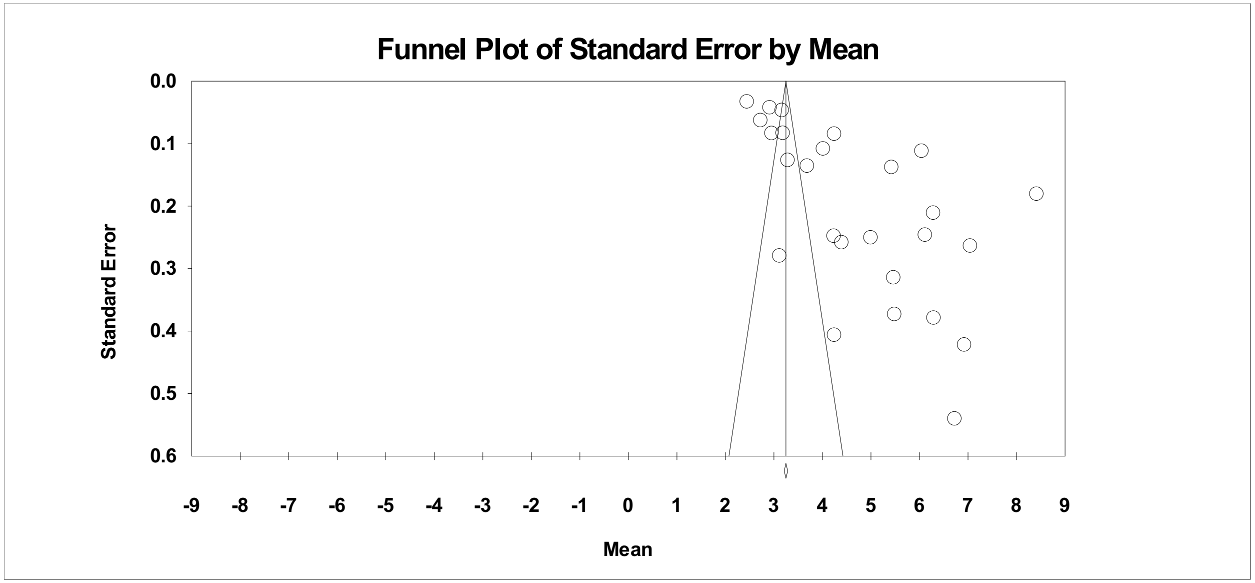

Figure S2. Funnel plot for mean NLR according to studies reported mortality (follow-up or in-hospital mortality).

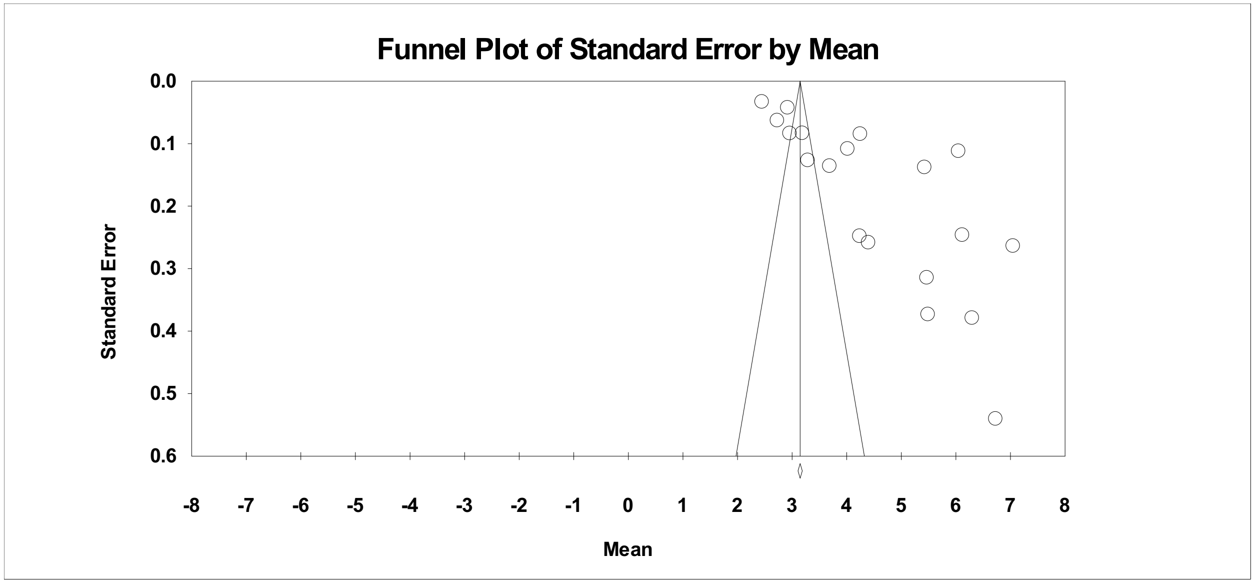

Figure S3. Funnel plot for mean NLR according to studies reported follow-up mortality.

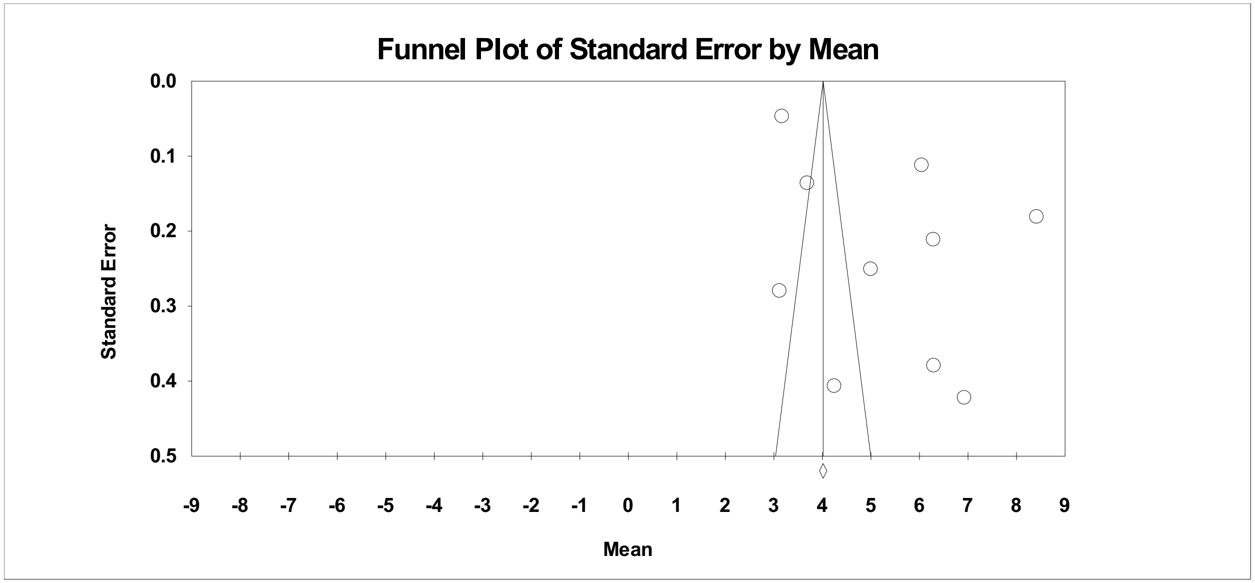

Figure S4. Funnel plot for mean NLR according to studies reported in-hospital mortality.

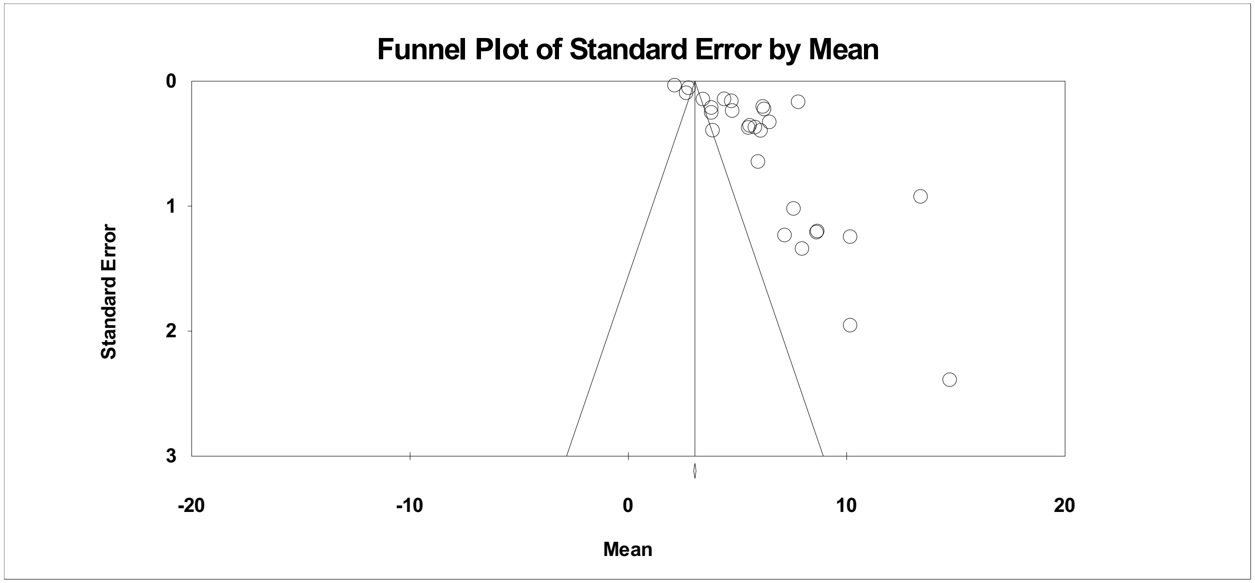

Figure S5. Funnel plot for mean NLR according to studies reported death or survived groups.

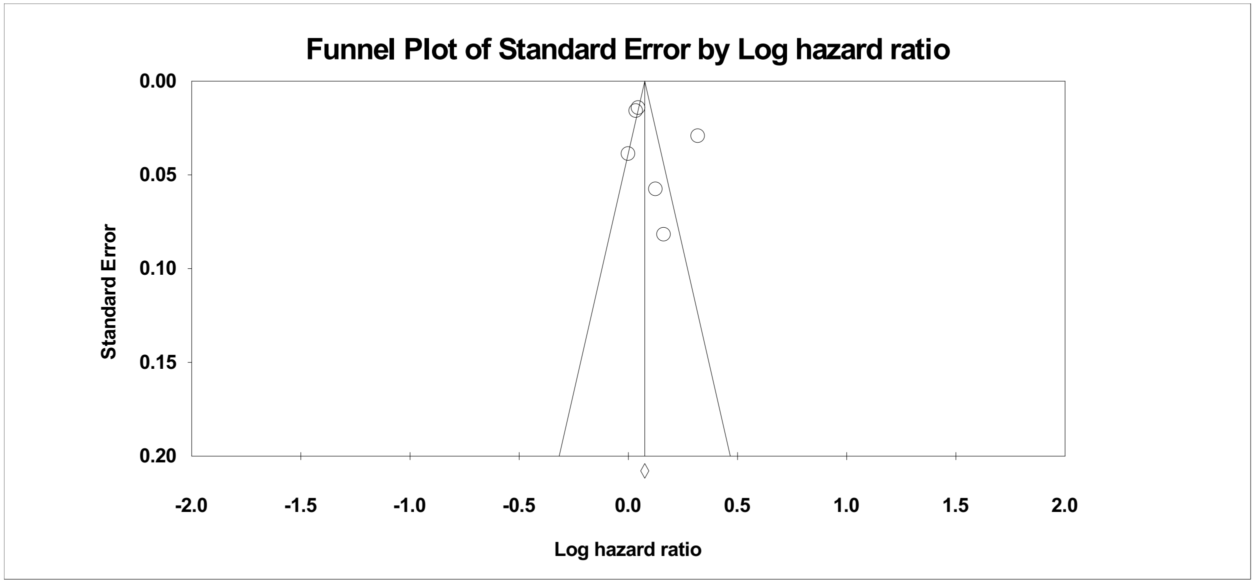

Figure S6. Funnel plot for NLR (as continuous variable) mortality hazard ratio.
